# How to apply an eye shield

**Published:** 2018-11-09

**Authors:** Nyawira Mwangi, Dorothy M Mutie

## Rationale

Applying an eye shield protects an injured eye from further damage.

## What you need

- Tape
- A rigid eye shield

If you do not have an eye shield, make one by cutting out a round piece of card approximately 8 cm in diameter. Make a single cut from the edge to the centre. Overlap the two edges and secure in place with tape to form a shallow cone (Figure 2).

## Instructions

1. Explain to the patient that the eye needs to be protected.
2. Ensure that there is good lighting.
3. Wash your hands.
4. Prepare the eye shield.
5. Ask the patient to close the affected eye.
6. Clean and dry the skin around the eye, as well as the forehead and cheek. This will allow the tape to hold fast.
7. Place the shield carefully over the eye. Ensure that the edges rest comfortably on the bones around the eye and not on the eye itself, or on the soft tissues surrounding it, as this can cause further damage.
8. Cut an appropriate length of tape (Figure 3).
9. Use the tape to hold the shield in place (Figure 4).

**Figure 1:**
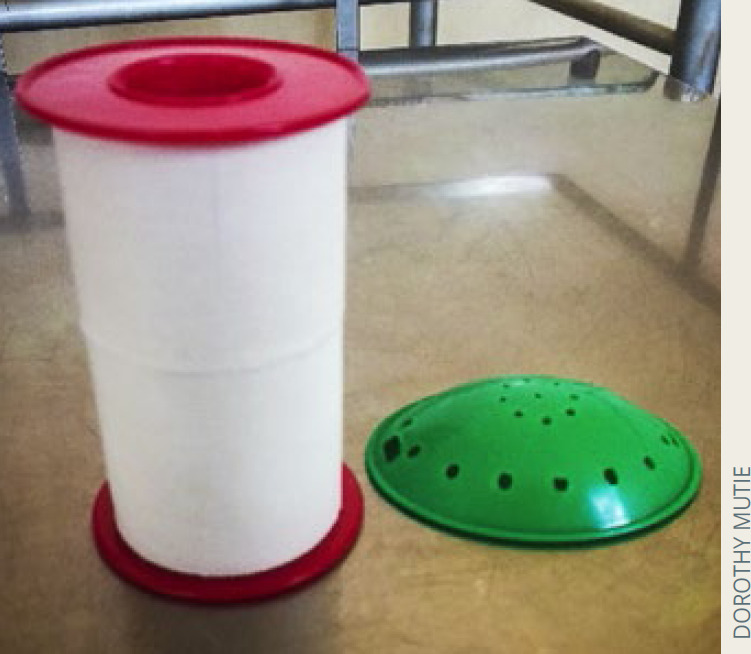
Rigid eye shield and tape

**Figure 2:**
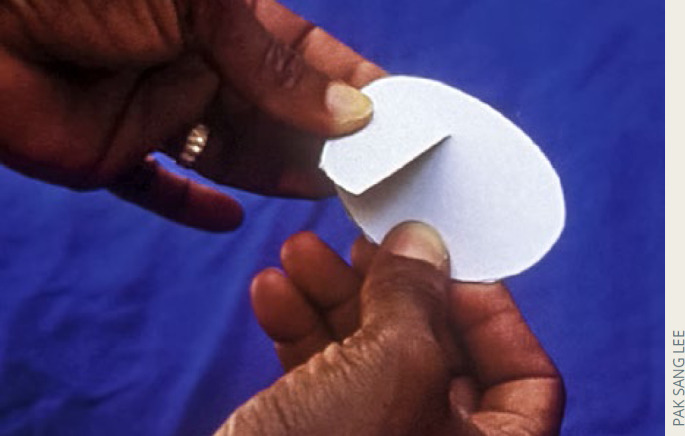
You can make your own eye shield using an oval piece of card

**Figure 3:**
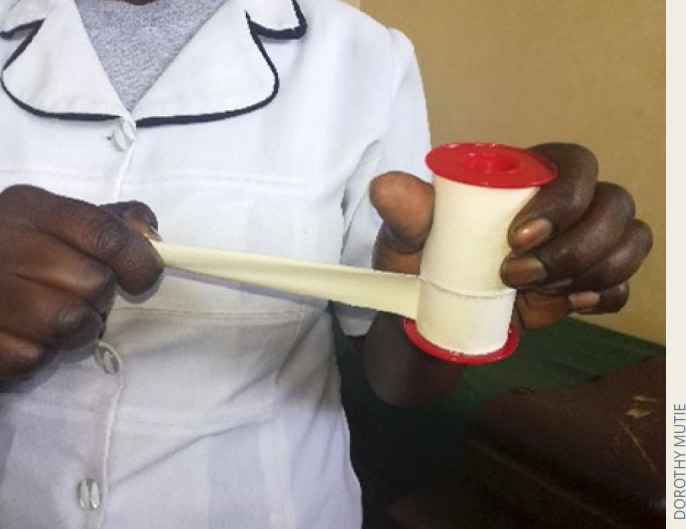
Cut an appropriate length of tape

**Figure 4:**
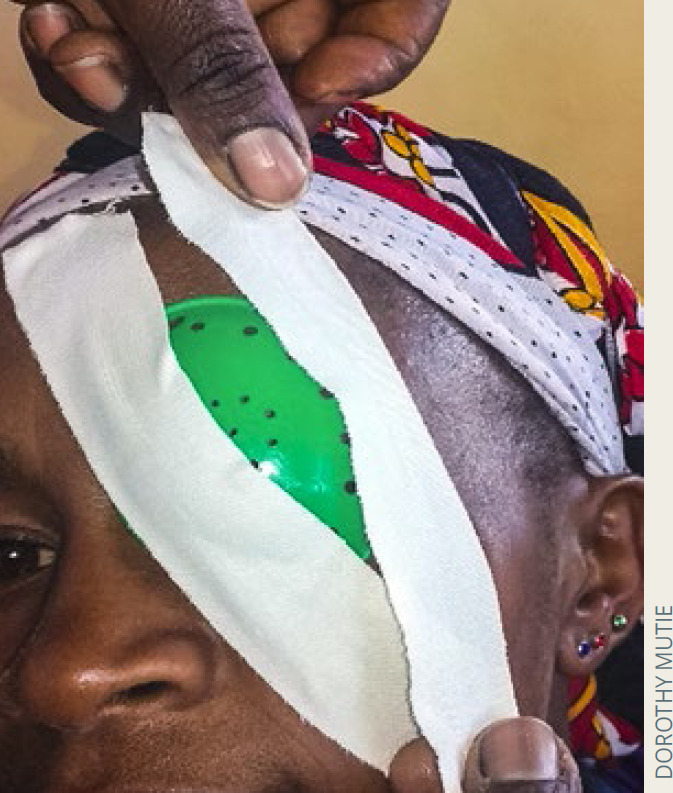
Apply tape to hold the shield in place

